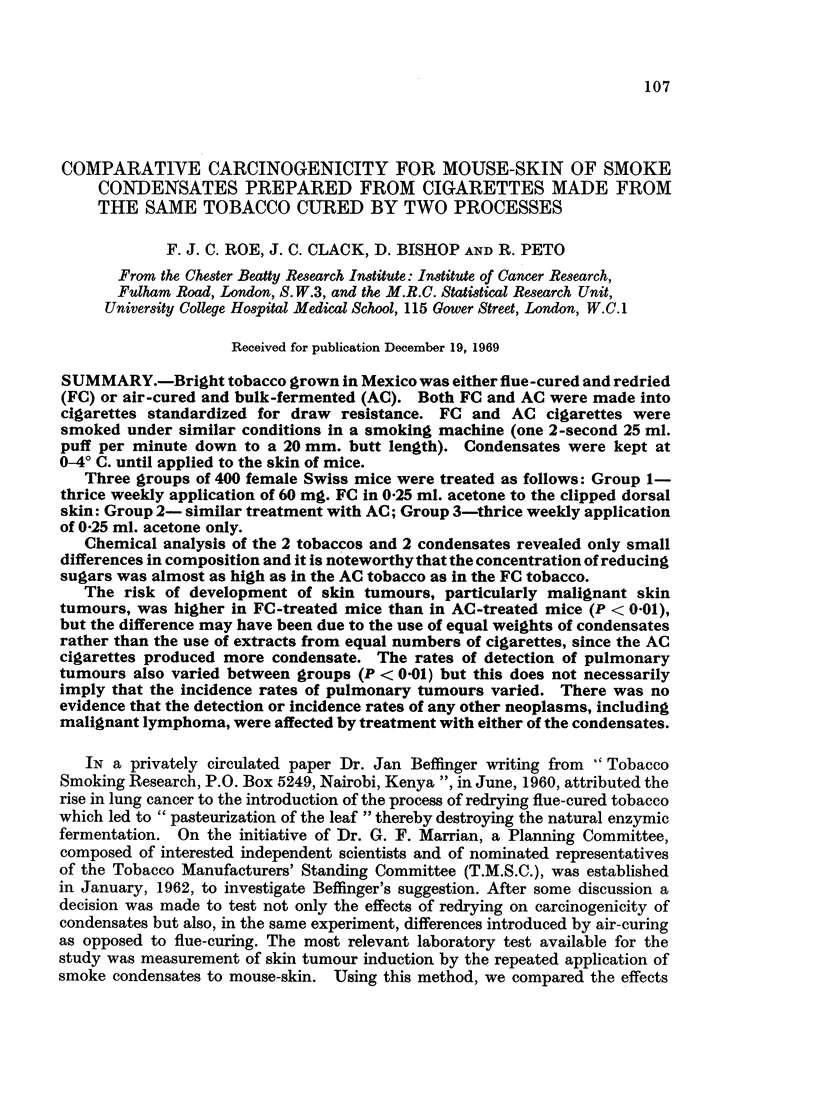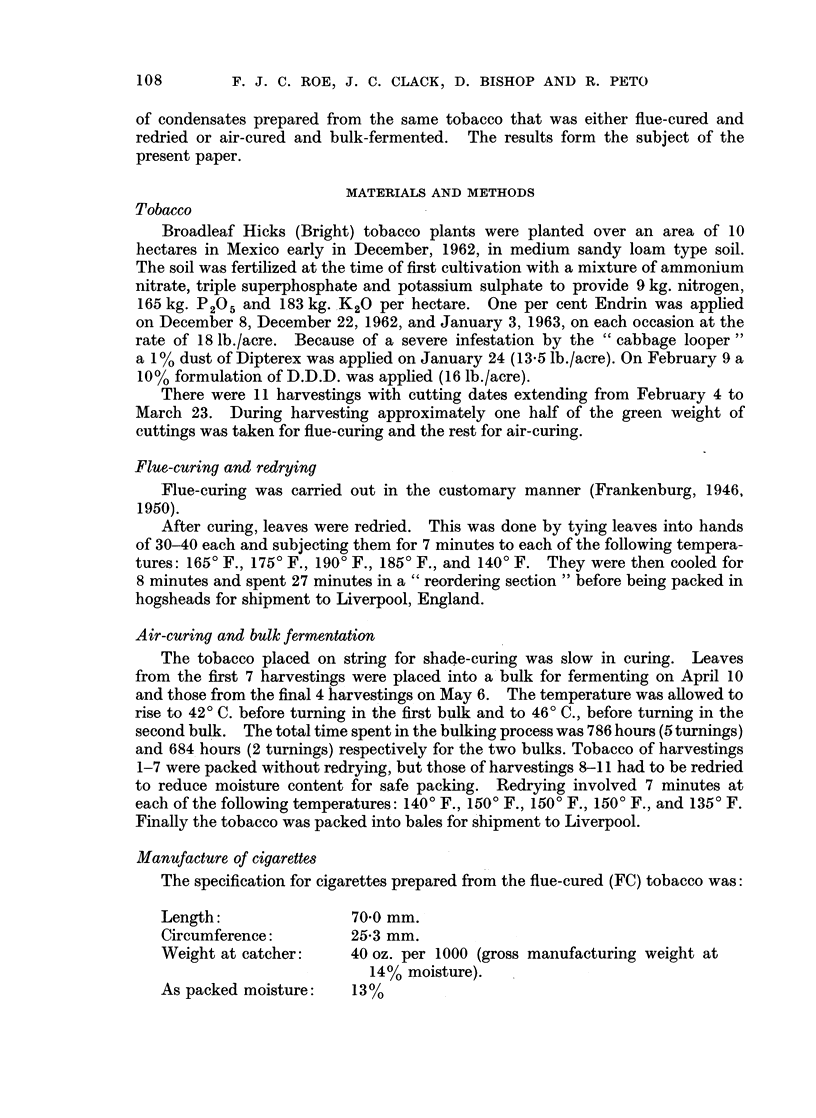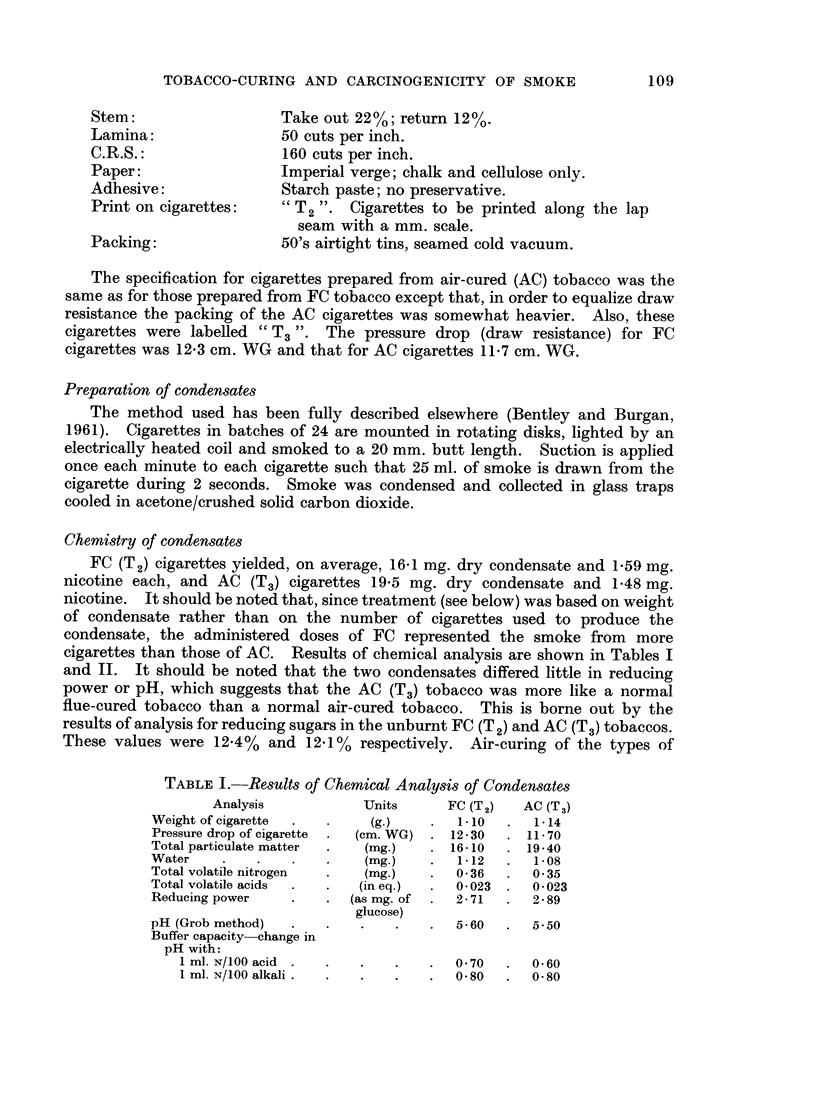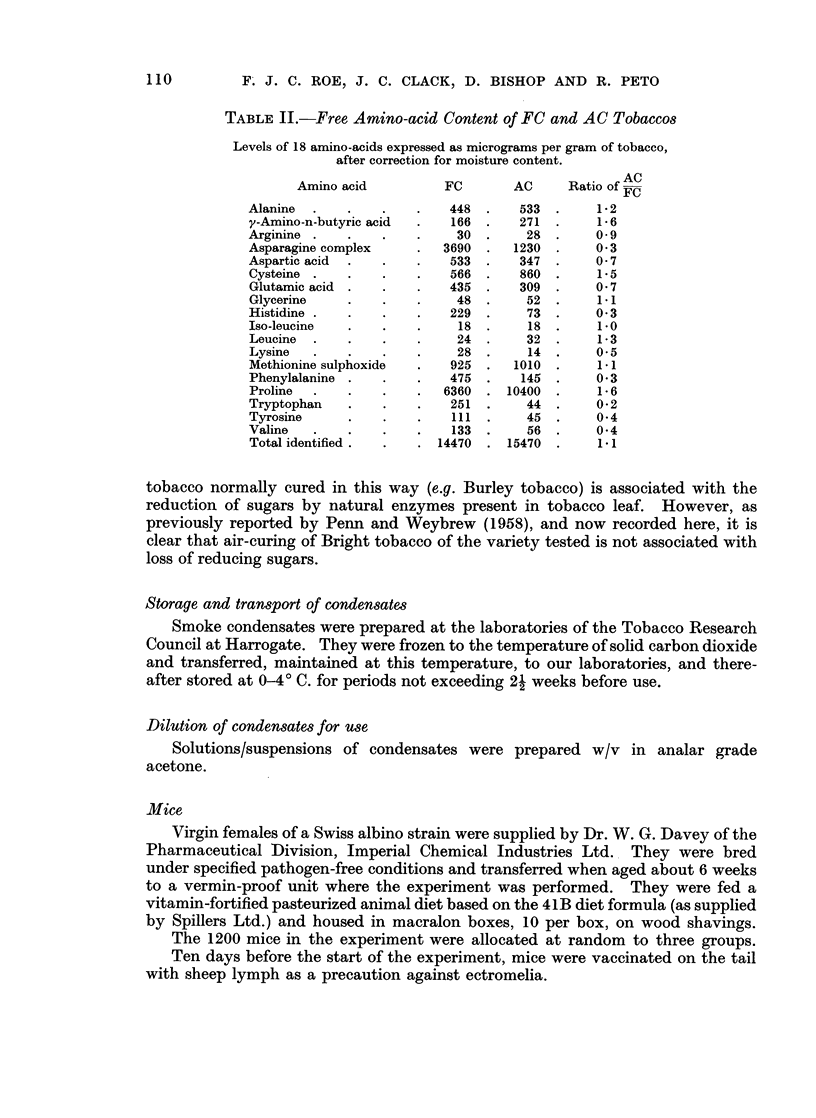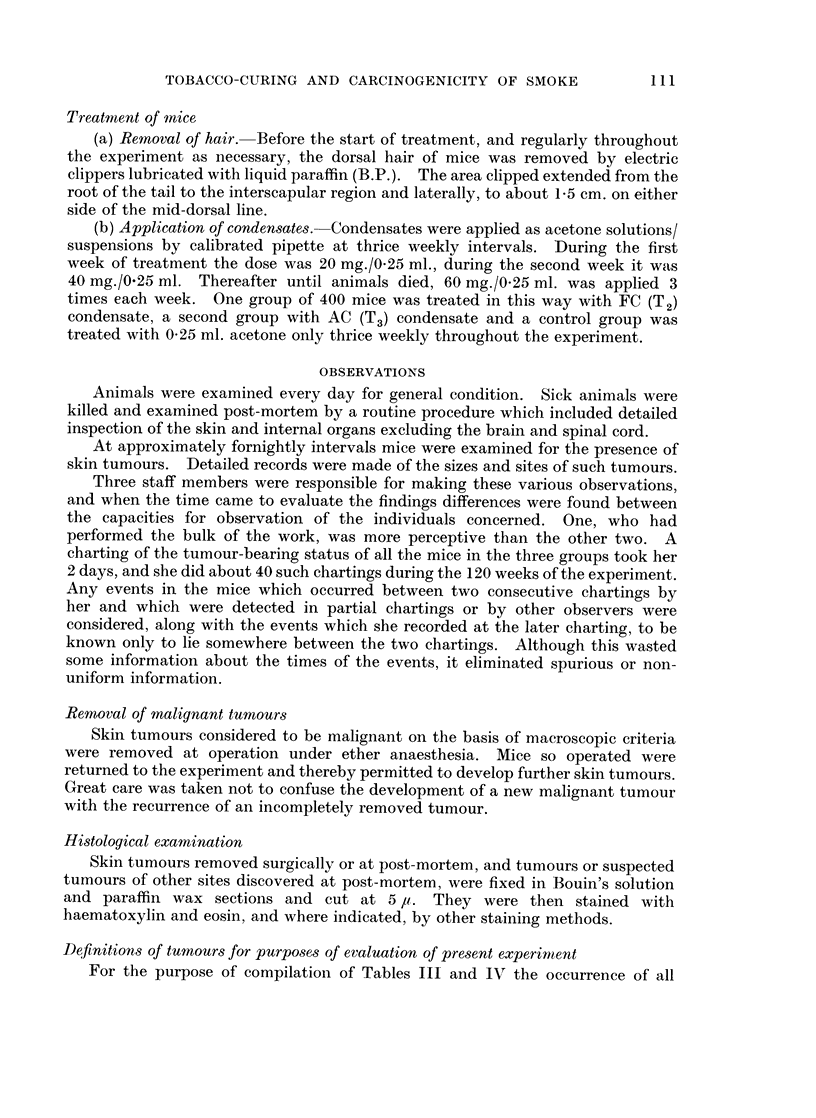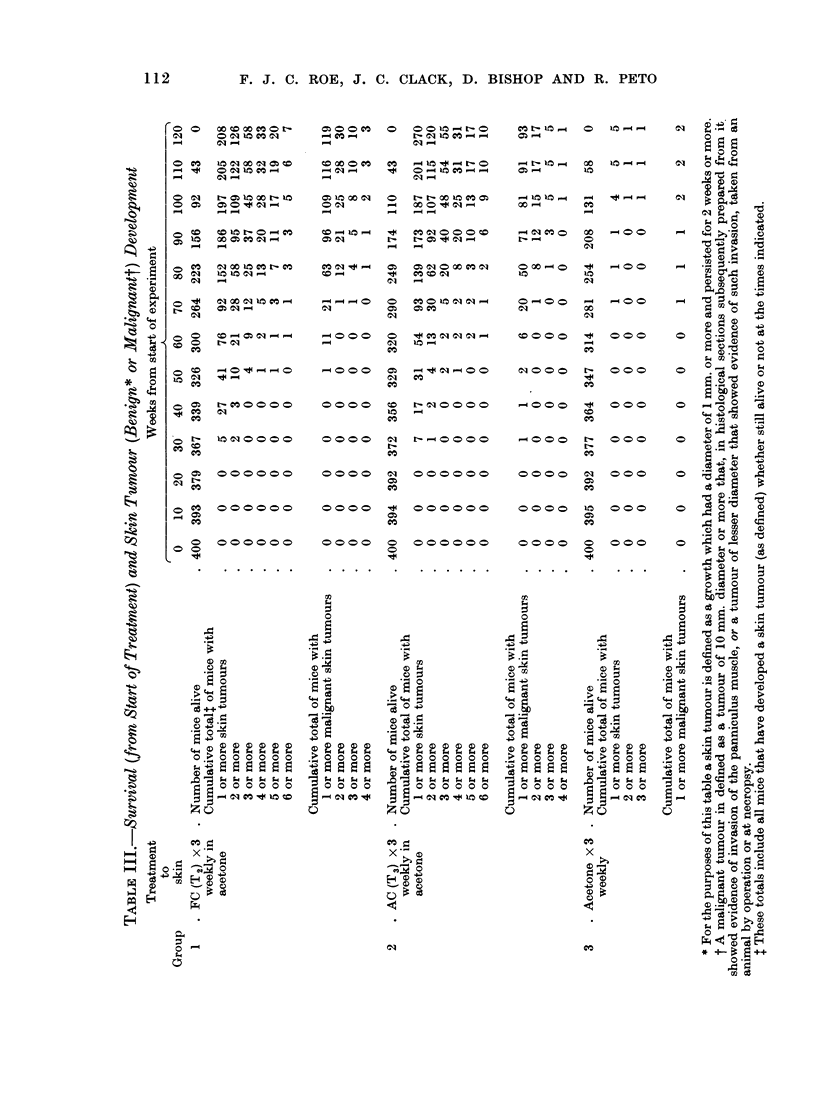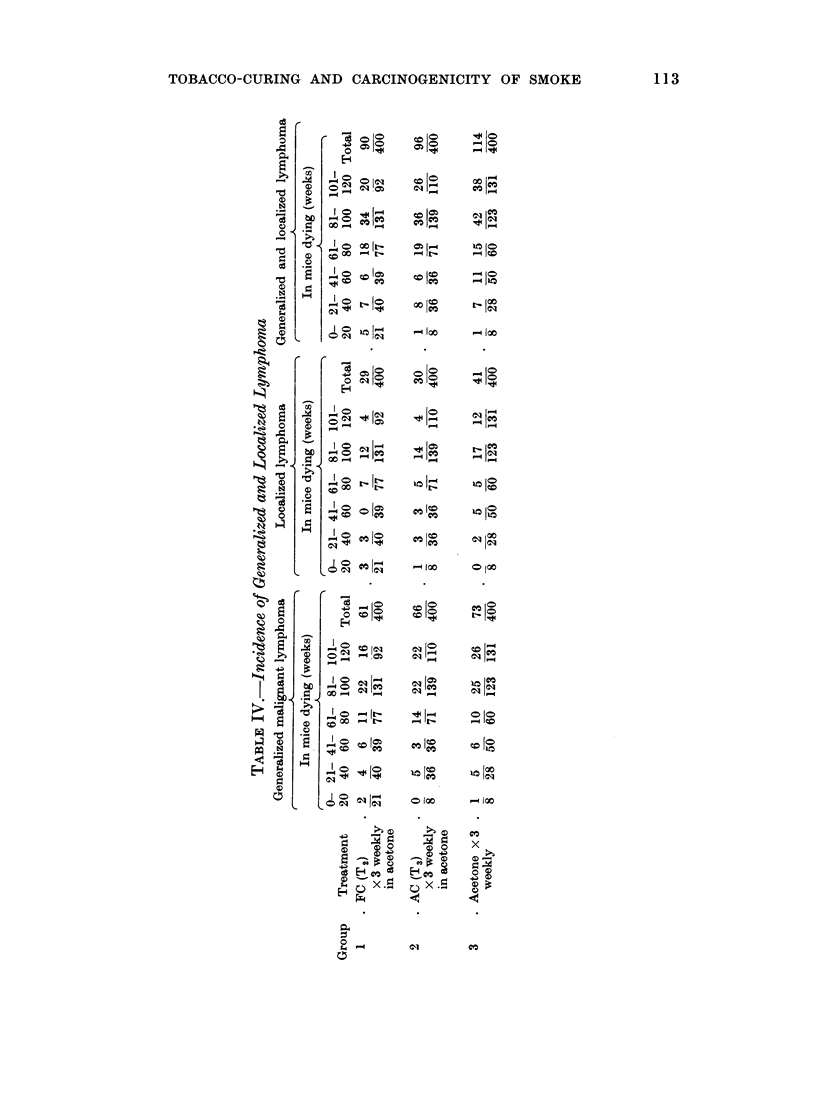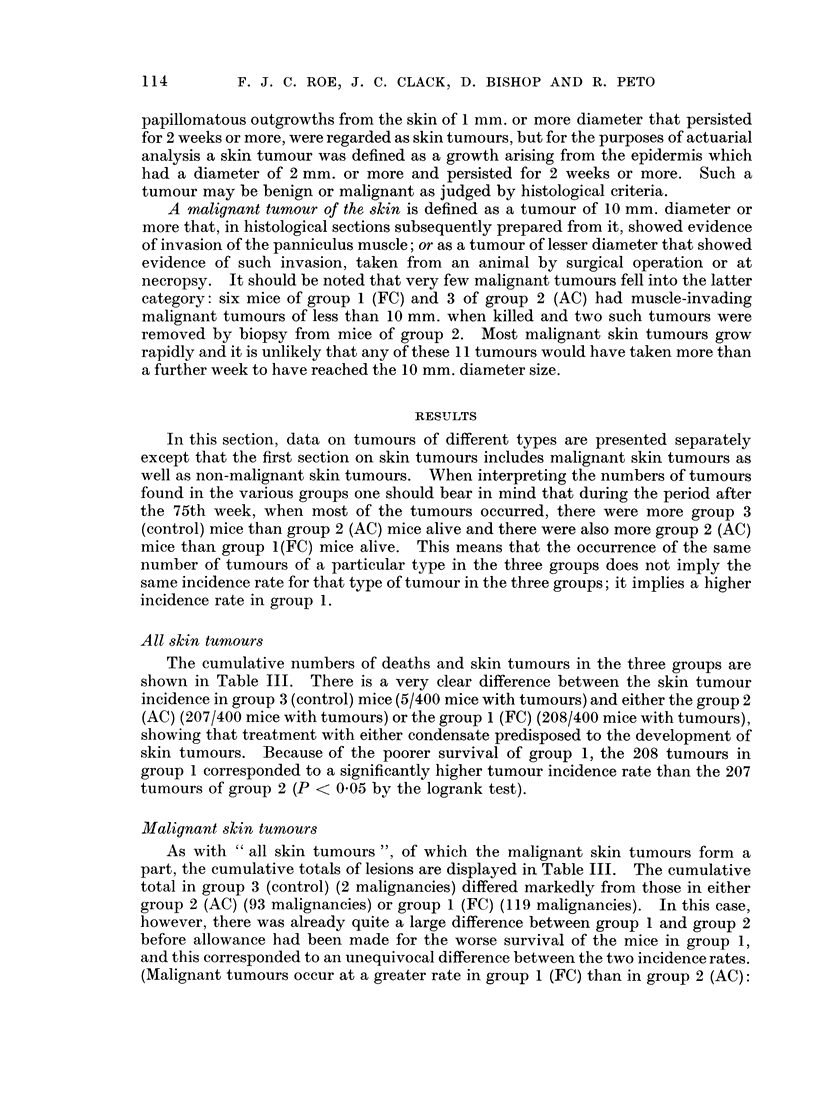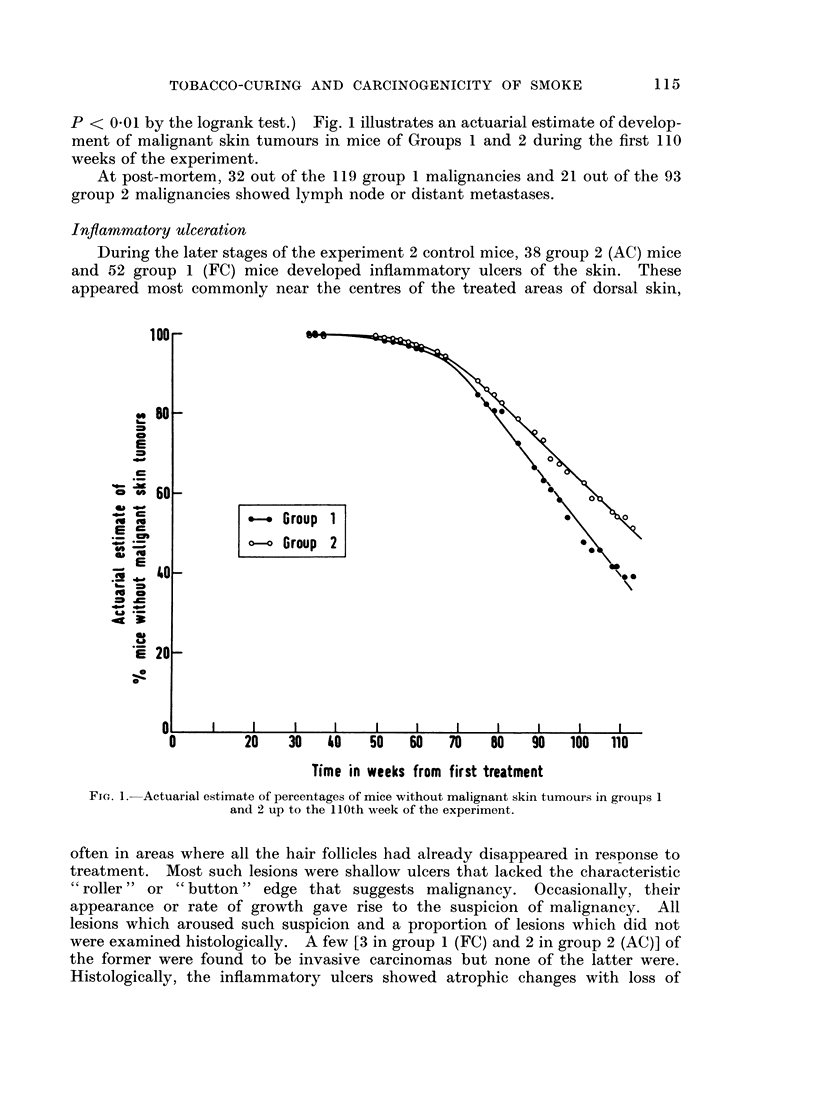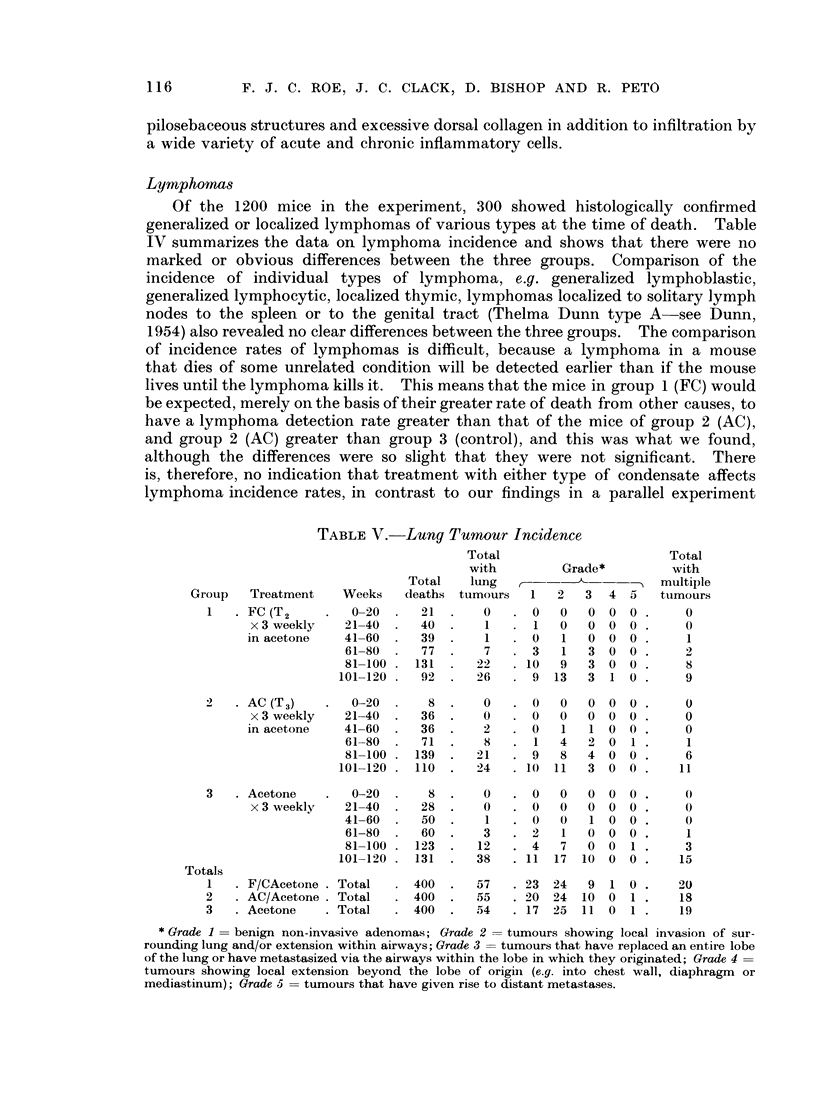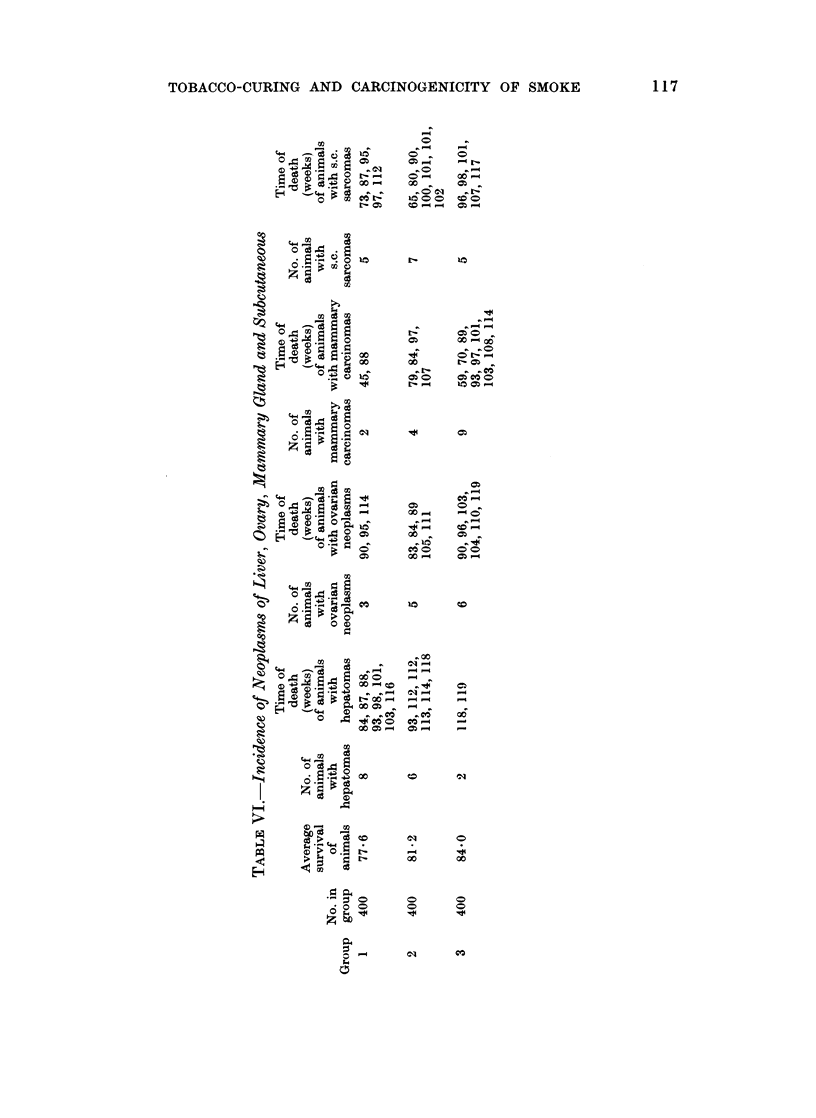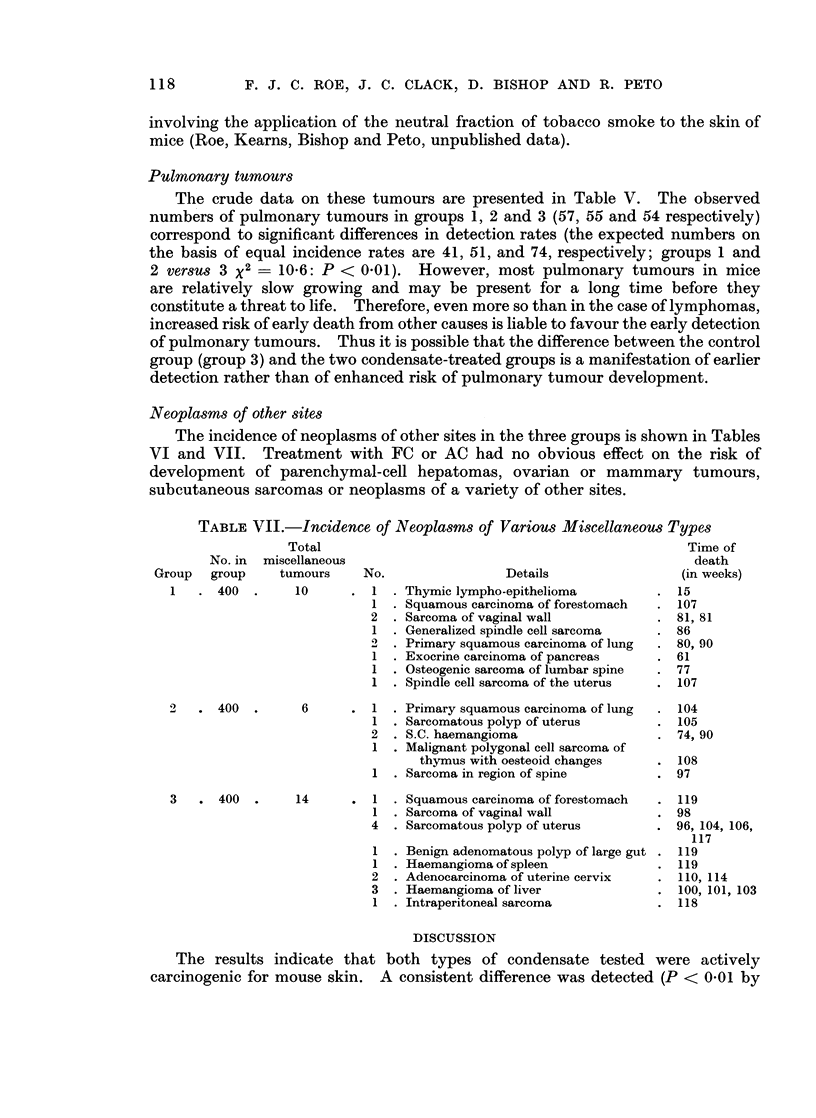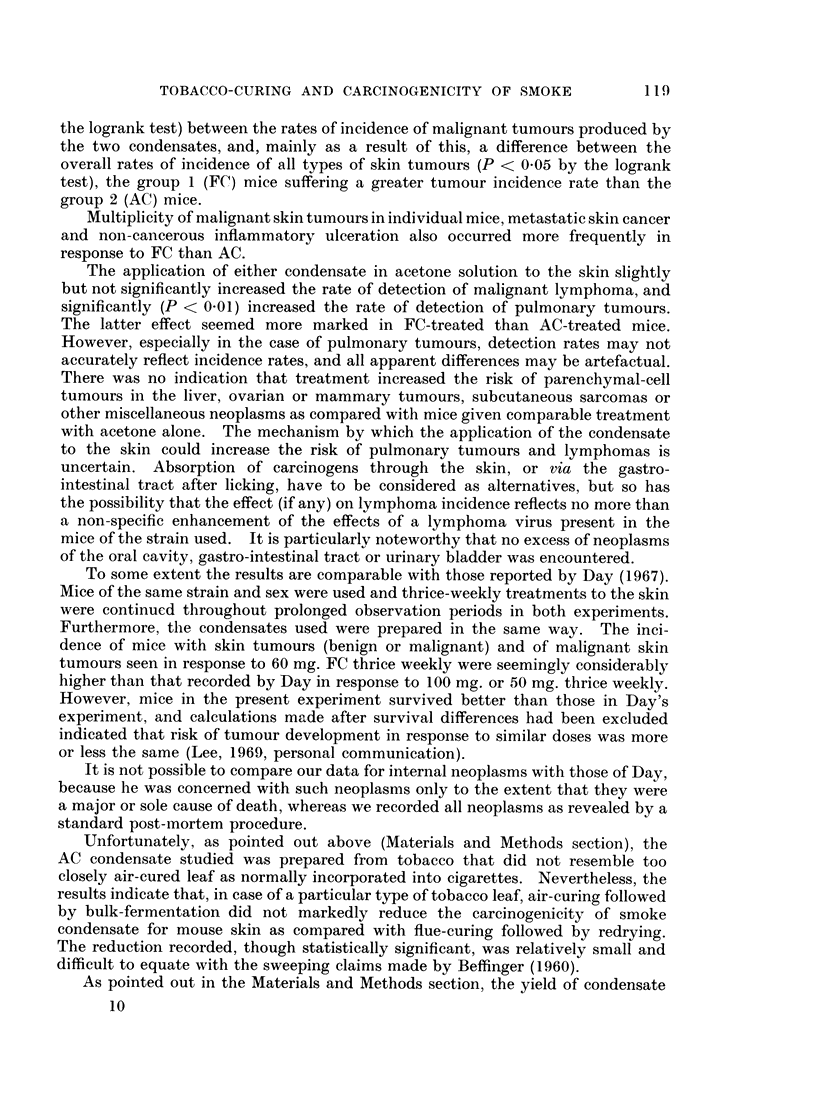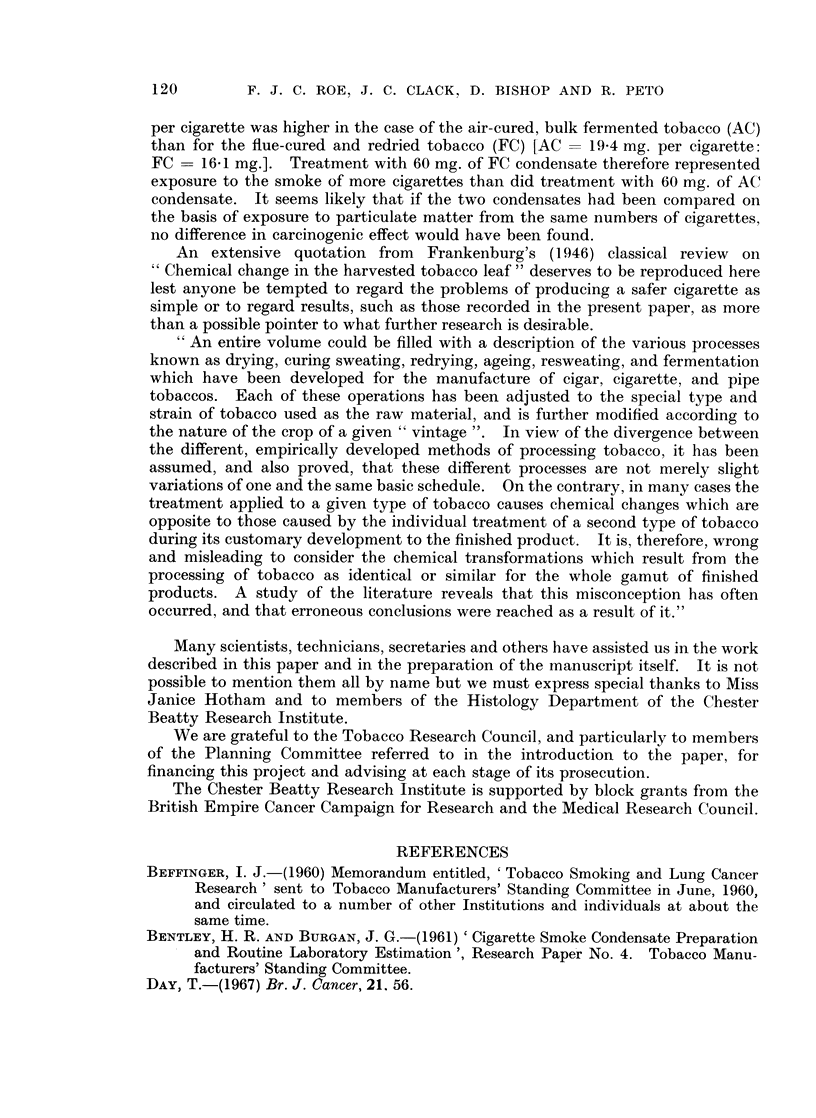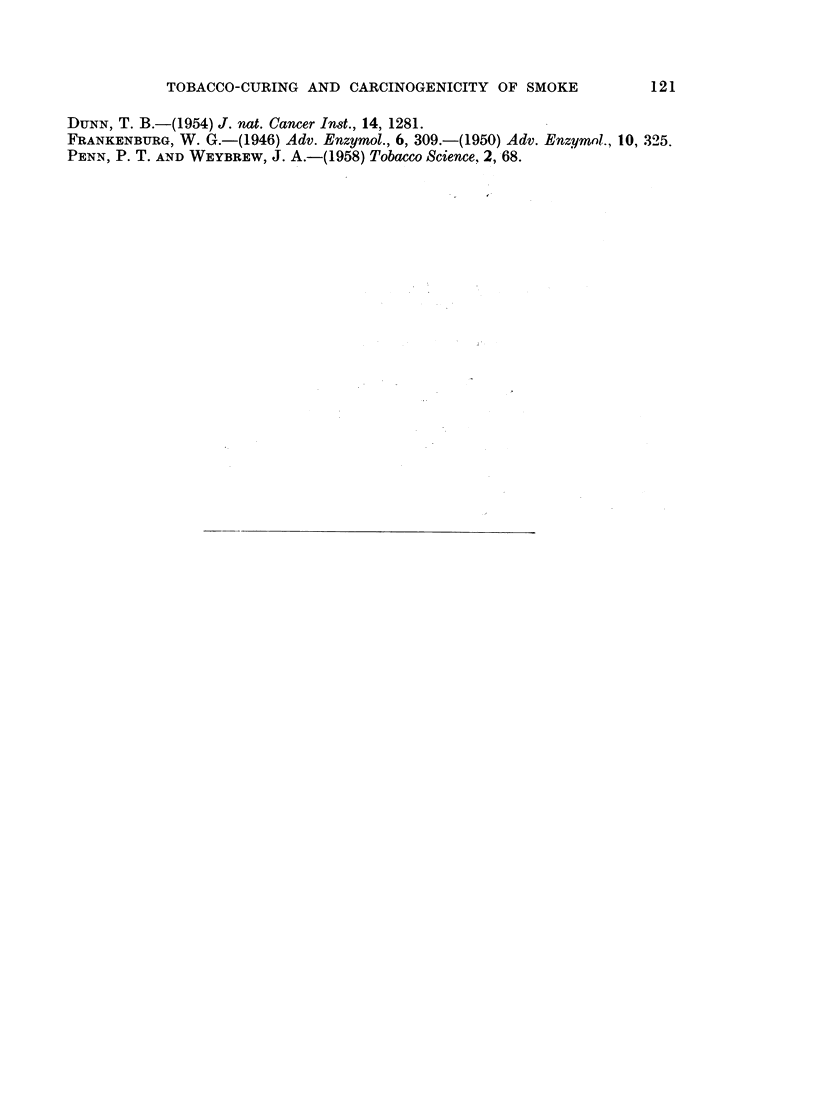# Comparative Carcinogenicity for Mouse-Skin of Smoke Condensates Prepared from Cigarettes Made from the Same Tobacco Cured by Two Processes

**DOI:** 10.1038/bjc.1970.14

**Published:** 1970-03

**Authors:** F. J. C. Roe, J. C. Clack, D. Bishop, R. Peto

## Abstract

Bright tobacco grown in Mexico was either flue-cured and redried (FC) or air-cured and bulk-fermented (AC). Both FC and AC were made into cigarettes standardized for draw resistance. FC and AC cigarettes were smoked under similar conditions in a smoking machine (one 2-second 25 ml. puff per minute down to a 20 mm. butt length). Condensates were kept at 0-4° C. until applied to the skin of mice.

Three groups of 400 female Swiss mice were treated as follows: Group 1— thrice weekly application of 60 mg. FC in 0.25 ml. acetone to the clipped dorsal skin: Group 2— similar treatment with AC; Group 3—thrice weekly application of 0.25 ml. acetone only.

Chemical analysis of the 2 tobaccos and 2 condensates revealed only small differences in composition and it is noteworthy that the concentration of reducing sugars was almost as high as in the AC tobacco as in the FC tobacco.

The risk of development of skin tumours, particularly malignant skin tumours, was higher in FC-treated mice than in AC-treated mice (p < 0.01), but the difference may have been due to the use of equal weights of condensates rather than the use of extracts from equal numbers of cigarettes, since the AC cigarettes produced more condensate. The rates of detection of pulmonary tumours also varied between groups (p < 0.01) but this does not necessarily imply that the incidence rates of pulmonary tumours varied. There was no evidence that the detection or incidence rates of any other neoplasms, including malignant lymphoma, were affected by treatment with either of the condensates.


### COMPARATIVE CARCINOGENICITY FOR MOUSE-SKIN OF SMOKE CONDENSATES PREPARED FROM CIGARETTES MADE FROM THE SAME TOBACCO CURED BY TWO PROCESSES

F. J. C. ROE, J. C. CLACK, D. BISHOP AND R. PETO

*From the Chester Beatty Research Institute: Institute of Cancer Research, Fulham Road, London, S.W.3, and the M.R.C. Statistical Research Unit, University College Hospital Medical School,* 115 *Gower Street, London, W.C.*1

Received for publication December 19, 1969

**SUMMARY.—Bright tobacco grown in Mexico was either flue-cured and redried (FC) or air-cured and bulk-fermented (AC). Both FC and AC were made into cigarettes standardized for draw resistance. FC and AC cigarettes were smoked under similar conditions in a smoking machine (one 2-second 25 ml. puff per minute down to a 20 mm. butt length). Condensates were kept at 0–4° C. until applied to the skin of mice.**

**Three groups of 400 female Swiss mice were treated as follows: Group 1—thrice weekly application of 60 mg. FC in 0·25 ml. acetone to the clipped dorsal skin: Group 2— similar treatment with AC; Group 3—thrice weekly application of 0·25 ml. acetone only.**

**Chemical analysis of the 2 tobaccos and 2 condensates revealed only small differences in composition and it is noteworthy that the concentration of reducing sugars was almost as high as in the AC tobacco as in the FC tobacco.**

**The risk of development of skin tumours, particularly malignant skin tumours, was higher in FC-treated mice than in AC-treated mice ($P < 0{\cdot}01$), but the difference may have been due to the use of equal weights of condensates rather than the use of extracts from equal numbers of cigarettes, since the AC cigarettes produced more condensate. The rates of detection of pulmonary tumours also varied between groups ($P < 0{\cdot}01$) but this does not necessarily imply that the incidence rates of pulmonary tumours varied. There was no evidence that the detection or incidence rates of any other neoplasms, including malignant lymphoma, were affected by treatment with either of the condensates.**

IN a privately circulated paper Dr. Jan Beffinger writing from " Tobacco Smoking Research, P.O. Box 5249, Nairobi, Kenya ", in June, 1960, attributed the rise in lung cancer to the introduction of the process of redrying flue-cured tobacco which led to " pasteurization of the leaf " thereby destroying the natural enzymic fermentation. On the initiative of Dr. G. F. Marrian, a Planning Committee, composed of interested independent scientists and of nominated representatives of the Tobacco Manufacturers' Standing Committee (T.M.S.C.), was established in January, 1962, to investigate Beffinger's suggestion. After some discussion a decision was made to test not only the effects of redrying on carcinogenicity of condensates but also, in the same experiment, differences introduced by air-curing as opposed to flue-curing. The most relevant laboratory test available for the study was measurement of skin tumour induction by the repeated application of smoke condensates to mouse-skin. Using this method, we compared the effects of condensates prepared from the same tobacco that was either flue-cured and redried or air-cured and bulk-fermented. The results form the subject of the present paper.

### MATERIALS AND METHODS

*Tobacco*

Broadleaf Hicks (Bright) tobacco plants were planted over an area of 10 hectares in Mexico early in December, 1962, in medium sandy loam type soil. The soil was fertilized at the time of first cultivation with a mixture of ammonium nitrate, triple superphosphate and potassium sulphate to provide 9 kg. nitrogen, 165 kg. $P_2O_5$ and 183 kg. $K_2O$ per hectare. One per cent Endrin was applied on December 8, December 22, 1962, and January 3, 1963, on each occasion at the rate of 18 lb./acre. Because of a severe infestation by the " cabbage looper " a 1% dust of Dipterex was applied on January 24 (13·5 lb./acre). On February 9 a 10% formulation of D.D.D. was applied (16 lb./acre).

There were 11 harvestings with cutting dates extending from February 4 to March 23. During harvesting approximately one half of the green weight of cuttings was taken for flue-curing and the rest for air-curing.

*Flue-curing and redrying*

Flue-curing was carried out in the customary manner (Frankenburg, 1946, 1950).

After curing, leaves were redried. This was done by tying leaves into hands of 30–40 each and subjecting them for 7 minutes to each of the following temperatures: 165° F., 175° F., 190° F., 185° F., and 140° F. They were then cooled for 8 minutes and spent 27 minutes in a " reordering section " before being packed in hogsheads for shipment to Liverpool, England.

*Air-curing and bulk fermentation*

The tobacco placed on string for shade-curing was slow in curing. Leaves from the first 7 harvestings were placed into a bulk for fermenting on April 10 and those from the final 4 harvestings on May 6. The temperature was allowed to rise to 42° C. before turning in the first bulk and to 46° C., before turning in the second bulk. The total time spent in the bulking process was 786 hours (5 turnings) and 684 hours (2 turnings) respectively for the two bulks. Tobacco of harvestings 1–7 were packed without redrying, but those of harvestings 8–11 had to be redried to reduce moisture content for safe packing. Redrying involved 7 minutes at each of the following temperatures: 140° F., 150° F., 150° F., 150° F., and 135° F. Finally the tobacco was packed into bales for shipment to Liverpool.

*Manufacture of cigarettes*

The specification for cigarettes prepared from the flue-cured (FC) tobacco was:

| | |
|---|---|
| Length: | 70·0 mm. |
| Circumference: | 25·3 mm. |
| Weight at catcher: | 40 oz. per 1000 (gross manufacturing weight at 14% moisture). |
| As packed moisture: | 13% |

| | |
|---|---|
| Stem: | Take out 22%; return 12%. |
| Lamina: | 50 cuts per inch. |
| C.R.S.: | 160 cuts per inch. |
| Paper: | Imperial verge; chalk and cellulose only. |
| Adhesive: | Starch paste; no preservative. |
| Print on cigarettes: | " $T_2$ ". Cigarettes to be printed along the lap seam with a mm. scale. |
| Packing: | 50's airtight tins, seamed cold vacuum. |

The specification for cigarettes prepared from air-cured (AC) tobacco was the same as for those prepared from FC tobacco except that, in order to equalize draw resistance the packing of the AC cigarettes was somewhat heavier. Also, these cigarettes were labelled " $T_3$ ". The pressure drop (draw resistance) for FC cigarettes was 12·3 cm. WG and that for AC cigarettes 11·7 cm. WG.

*Preparation of condensates*

The method used has been fully described elsewhere (Bentley and Burgan, 1961). Cigarettes in batches of 24 are mounted in rotating disks, lighted by an electrically heated coil and smoked to a 20 mm. butt length. Suction is applied once each minute to each cigarette such that 25 ml. of smoke is drawn from the cigarette during 2 seconds. Smoke was condensed and collected in glass traps cooled in acetone/crushed solid carbon dioxide.

*Chemistry of condensates*

FC ($T_2$) cigarettes yielded, on average, 16·1 mg. dry condensate and 1·59 mg. nicotine each, and AC ($T_3$) cigarettes 19·5 mg. dry condensate and 1·48 mg. nicotine. It should be noted that, since treatment (see below) was based on weight of condensate rather than on the number of cigarettes used to produce the condensate, the administered doses of FC represented the smoke from more cigarettes than those of AC. Results of chemical analysis are shown in Tables I and II. It should be noted that the two condensates differed little in reducing power or pH, which suggests that the AC ($T_3$) tobacco was more like a normal flue-cured tobacco than a normal air-cured tobacco. This is borne out by the results of analysis for reducing sugars in the unburnt FC ($T_2$) and AC ($T_3$) tobaccos. These values were 12·4% and 12·1% respectively. Air-curing of the types of

TABLE I.—*Results of Chemical Analysis of Condensates*

| Analysis | Units | FC ($T_2$) | AC ($T_3$) |
|---|---|---|---|
| Weight of cigarette | (g.) | 1·10 | 1·14 |
| Pressure drop of cigarette | (cm. WG) | 12·30 | 11·70 |
| Total particulate matter | (mg.) | 16·10 | 19·40 |
| Water | (mg.) | 1·12 | 1·08 |
| Total volatile nitrogen | (mg.) | 0·36 | 0·35 |
| Total volatile acids | (in eq.) | 0·023 | 0·023 |
| Reducing power | (as mg. of glucose) | 2·71 | 2·89 |
| pH (Grob method) | | 5·60 | 5·50 |
| Buffer capacity—change in pH with: | | | |
| 1 ml. N/100 acid | | 0·70 | 0·60 |
| 1 ml. N/100 alkali | | 0·80 | 0·80 |

TABLE II.—*Free Amino-acid Content of FC and AC Tobaccos*

Levels of 18 amino-acids expressed as micrograms per gram of tobacco, after correction for moisture content.

| Amino acid | FC | AC | Ratio of $\frac{AC}{FC}$ |
|---|---|---|---|
| Alanine | 448 | 533 | 1·2 |
| γ-Amino-n-butyric acid | 166 | 271 | 1·6 |
| Arginine | 30 | 28 | 0·9 |
| Asparagine complex | 3690 | 1230 | 0·3 |
| Aspartic acid | 533 | 347 | 0·7 |
| Cysteine | 566 | 860 | 1·5 |
| Glutamic acid | 435 | 309 | 0·7 |
| Glycerine | 48 | 52 | 1·1 |
| Histidine | 229 | 73 | 0·3 |
| Iso-leucine | 18 | 18 | 1·0 |
| Leucine | 24 | 32 | 1·3 |
| Lysine | 28 | 14 | 0·5 |
| Methionine sulphoxide | 925 | 1010 | 1·1 |
| Phenylalanine | 475 | 145 | 0·3 |
| Proline | 6360 | 10400 | 1·6 |
| Tryptophan | 251 | 44 | 0·2 |
| Tyrosine | 111 | 45 | 0·4 |
| Valine | 133 | 56 | 0·4 |
| Total identified | 14470 | 15470 | 1·1 |

tobacco normally cured in this way (*e.g.* Burley tobacco) is associated with the reduction of sugars by natural enzymes present in tobacco leaf. However, as previously reported by Penn and Weybrew (1958), and now recorded here, it is clear that air-curing of Bright tobacco of the variety tested is not associated with loss of reducing sugars.

*Storage and transport of condensates*

Smoke condensates were prepared at the laboratories of the Tobacco Research Council at Harrogate. They were frozen to the temperature of solid carbon dioxide and transferred, maintained at this temperature, to our laboratories, and thereafter stored at 0–4° C. for periods not exceeding 2½ weeks before use.

*Dilution of condensates for use*

Solutions/suspensions of condensates were prepared w/v in analar grade acetone.

*Mice*

Virgin females of a Swiss albino strain were supplied by Dr. W. G. Davey of the Pharmaceutical Division, Imperial Chemical Industries Ltd. They were bred under specified pathogen-free conditions and transferred when aged about 6 weeks to a vermin-proof unit where the experiment was performed. They were fed a vitamin-fortified pasteurized animal diet based on the 41B diet formula (as supplied by Spillers Ltd.) and housed in macralon boxes, 10 per box, on wood shavings.

The 1200 mice in the experiment were allocated at random to three groups.

Ten days before the start of the experiment, mice were vaccinated on the tail with sheep lymph as a precaution against ectromelia.

*Treatment of mice*

(a) *Removal of hair.*—Before the start of treatment, and regularly throughout the experiment as necessary, the dorsal hair of mice was removed by electric clippers lubricated with liquid paraffin (B.P.). The area clipped extended from the root of the tail to the interscapular region and laterally, to about 1·5 cm. on either side of the mid-dorsal line.

(b) *Application of condensates.*—Condensates were applied as acetone solutions/suspensions by calibrated pipette at thrice weekly intervals. During the first week of treatment the dose was 20 mg./0·25 ml., during the second week it was 40 mg./0·25 ml. Thereafter until animals died, 60 mg./0·25 ml. was applied 3 times each week. One group of 400 mice was treated in this way with FC ($T_2$) condensate, a second group with AC ($T_3$) condensate and a control group was treated with 0·25 ml. acetone only thrice weekly throughout the experiment.

OBSERVATIONS

Animals were examined every day for general condition. Sick animals were killed and examined post-mortem by a routine procedure which included detailed inspection of the skin and internal organs excluding the brain and spinal cord.

At approximately fornightly intervals mice were examined for the presence of skin tumours. Detailed records were made of the sizes and sites of such tumours.

Three staff members were responsible for making these various observations, and when the time came to evaluate the findings differences were found between the capacities for observation of the individuals concerned. One, who had performed the bulk of the work, was more perceptive than the other two. A charting of the tumour-bearing status of all the mice in the three groups took her 2 days, and she did about 40 such chartings during the 120 weeks of the experiment. Any events in the mice which occurred between two consecutive chartings by her and which were detected in partial chartings or by other observers were considered, along with the events which she recorded at the later charting, to be known only to lie somewhere between the two chartings. Although this wasted some information about the times of the events, it eliminated spurious or non-uniform information.

*Removal of malignant tumours*

Skin tumours considered to be malignant on the basis of macroscopic criteria were removed at operation under ether anaesthesia. Mice so operated were returned to the experiment and thereby permitted to develop further skin tumours. Great care was taken not to confuse the development of a new malignant tumour with the recurrence of an incompletely removed tumour.

*Histological examination*

Skin tumours removed surgically or at post-mortem, and tumours or suspected tumours of other sites discovered at post-mortem, were fixed in Bouin's solution and paraffin wax sections and cut at 5 $\mu$. They were then stained with haematoxylin and eosin, and where indicated, by other staining methods.

*Definitions of tumours for purposes of evaluation of present experiment*

For the purpose of compilation of Tables III and IV the occurrence of all

TABLE III.—*Survival (from Start of Treatment) and Skin Tumour (Benign* or Malignant†) Development*

| Group | Treatment to skin | | Weeks from start of experiment: 0 | 10 | 20 | 30 | 40 | 50 | 60 | 70 | 80 | 90 | 100 | 110 | 120 |
|---|---|---|---|---|---|---|---|---|---|---|---|---|---|---|---|
| 1 | FC ($T_2$) ×3 weekly in acetone | Number of mice alive | 400 | 393 | 379 | 367 | 339 | 326 | 300 | 264 | 223 | 156 | 92 | 43 | 0 |
| | | Cumulative total‡ of mice with | | | | | | | | | | | | | |
| | | 1 or more skin tumours | 0 | 0 | 0 | 5 | 27 | 41 | 76 | 92 | 152 | 186 | 197 | 205 | 208 |
| | | 2 or more | 0 | 0 | 0 | 2 | 3 | 10 | 21 | 28 | 58 | 95 | 109 | 122 | 126 |
| | | 3 or more | 0 | 0 | 0 | 0 | 0 | 4 | 9 | 12 | 25 | 37 | 45 | 58 | 58 |
| | | 4 or more | 0 | 0 | 0 | 0 | 0 | 1 | 2 | 5 | 13 | 20 | 28 | 32 | 33 |
| | | 5 or more | 0 | 0 | 0 | 0 | 0 | 1 | 1 | 3 | 7 | 11 | 17 | 19 | 20 |
| | | 6 or more | 0 | 0 | 0 | 0 | 0 | 0 | 1 | 1 | 3 | 3 | 5 | 6 | 7 |
| | | Cumulative total of mice with | | | | | | | | | | | | | |
| | | 1 or more malignant skin tumours | 0 | 0 | 0 | 0 | 0 | 1 | 11 | 21 | 63 | 96 | 109 | 116 | 119 |
| | | 2 or more | 0 | 0 | 0 | 0 | 0 | 0 | 0 | 1 | 12 | 21 | 25 | 28 | 30 |
| | | 3 or more | 0 | 0 | 0 | 0 | 0 | 0 | 0 | 1 | 4 | 5 | 8 | 10 | 10 |
| | | 4 or more | 0 | 0 | 0 | 0 | 0 | 0 | 0 | 0 | 1 | 1 | 2 | 3 | 3 |
| 2 | AC ($T_3$) ×3 weekly in acetone | Number of mice alive | 400 | 394 | 392 | 372 | 356 | 329 | 320 | 290 | 249 | 174 | 110 | 43 | 0 |
| | | Cumulative total of mice with | | | | | | | | | | | | | |
| | | 1 or more skin tumours | 0 | 0 | 0 | 7 | 17 | 31 | 54 | 93 | 139 | 173 | 187 | 201 | 270 |
| | | 2 or more | 0 | 0 | 0 | 1 | 2 | 4 | 13 | 30 | 62 | 92 | 107 | 115 | 120 |
| | | 3 or more | 0 | 0 | 0 | 0 | 0 | 2 | 2 | 5 | 20 | 40 | 48 | 54 | 55 |
| | | 4 or more | 0 | 0 | 0 | 0 | 0 | 1 | 2 | 2 | 8 | 20 | 25 | 31 | 31 |
| | | 5 or more | 0 | 0 | 0 | 0 | 0 | 0 | 2 | 2 | 3 | 10 | 13 | 17 | 17 |
| | | 6 or more | 0 | 0 | 0 | 0 | 0 | 0 | 1 | 1 | 2 | 6 | 9 | 10 | 10 |
| | | Cumulative total of mice with | | | | | | | | | | | | | |
| | | 1 or more malignant skin tumours | 0 | 0 | 0 | 1 | 1 | 2 | 6 | 20 | 50 | 71 | 81 | 91 | 93 |
| | | 2 or more | 0 | 0 | 0 | 0 | 0 | 0 | 0 | 1 | 8 | 12 | 15 | 17 | 17 |
| | | 3 or more | 0 | 0 | 0 | 0 | 0 | 0 | 0 | 0 | 1 | 3 | 5 | 5 | 5 |
| | | 4 or more | 0 | 0 | 0 | 0 | 0 | 0 | 0 | 0 | 0 | 0 | 1 | 1 | 1 |
| 3 | Acetone ×3 weekly | Number of mice alive | 400 | 395 | 392 | 377 | 364 | 347 | 314 | 281 | 254 | 208 | 131 | 58 | 0 |
| | | Cumulative total of mice with | | | | | | | | | | | | | |
| | | 1 or more skin tumours | 0 | 0 | 0 | 0 | 0 | 0 | 0 | 1 | 1 | 1 | 4 | 5 | 5 |
| | | 2 or more | 0 | 0 | 0 | 0 | 0 | 0 | 0 | 0 | 0 | 0 | 1 | 1 | 1 |
| | | 3 or more | 0 | 0 | 0 | 0 | 0 | 0 | 0 | 0 | 0 | 0 | 1 | 1 | 1 |
| | | Cumulative total of mice with | | | | | | | | | | | | | |
| | | 1 or more malignant skin tumours | 0 | 0 | 0 | 0 | 0 | 0 | 0 | 1 | 1 | 1 | 2 | 2 | 2 |

* For the purposes of this table a skin tumour is defined as a growth which had a diameter of 1 mm. or more and persisted for 2 weeks or more.

† A malignant tumour in defined as a tumour of 10 mm. diameter or more that, in histological sections subsequently prepared from it showed evidence of invasion of the panniculus muscle, *or* a tumour of lesser diameter that showed evidence of such invasion, taken from an animal by operation or at necropsy.

‡ These totals include all mice that have developed a skin tumour (as defined) whether still alive or not at the times indicated.

TABLE IV.—*Incidence of Generalized and Localized Lymphoma*

| Group | Treatment | Generalized malignant lymphoma: In mice dying (weeks) | | | | | | | Localized lymphoma: In mice dying (weeks) | | | | | | | Generalized and localized lymphoma: In mice dying (weeks) | | | | | | |
|---|---|---|---|---|---|---|---|---|---|---|---|---|---|---|---|---|---|---|---|---|---|---|
| | | 0–20 | 21–40 | 41–60 | 61–80 | 81–100 | 101–120 | Total | 0–20 | 21–40 | 41–60 | 61–80 | 81–100 | 101–120 | Total | 0–20 | 21–40 | 41–60 | 61–80 | 81–100 | 101–120 | Total |
| 1 | FC ($T_2$) ×3 weekly in acetone | 2/21 | 4/40 | 6/39 | 11/77 | 22/131 | 16/92 | 61/400 | 3/21 | 3/40 | 0/39 | 7/77 | 12/131 | 4/92 | 29/400 | 5/21 | 7/40 | 6/39 | 18/77 | 34/131 | 20/92 | 90/400 |
| 2 | AC ($T_3$) ×3 weekly in acetone | 0/8 | 5/36 | 3/36 | 14/71 | 22/139 | 22/110 | 66/400 | 1/8 | 3/36 | 3/36 | 5/71 | 14/139 | 4/110 | 30/400 | 1/8 | 8/36 | 6/36 | 19/71 | 36/139 | 26/110 | 96/400 |
| 3 | Acetone ×3 weekly | 1/8 | 5/28 | 6/50 | 10/60 | 25/123 | 26/131 | 73/400 | 0/8 | 2/28 | 5/50 | 5/60 | 17/123 | 12/131 | 41/400 | 1/8 | 7/28 | 11/50 | 15/60 | 42/123 | 38/131 | 114/400 |

papillomatous outgrowths from the skin of 1 mm. or more diameter that persisted for 2 weeks or more, were regarded as skin tumours, but for the purposes of actuarial analysis a skin tumour was defined as a growth arising from the epidermis which had a diameter of 2 mm. or more and persisted for 2 weeks or more. Such a tumour may be benign or malignant as judged by histological criteria.

*A malignant tumour of the skin* is defined as a tumour of 10 mm. diameter or more that, in histological sections subsequently prepared from it, showed evidence of invasion of the panniculus muscle; *or* as a tumour of lesser diameter that showed evidence of such invasion, taken from an animal by surgical operation or at necropsy. It should be noted that very few malignant tumours fell into the latter category: six mice of group 1 (FC) and 3 of group 2 (AC) had muscle-invading malignant tumours of less than 10 mm. when killed and two such tumours were removed by biopsy from mice of group 2. Most malignant skin tumours grow rapidly and it is unlikely that any of these 11 tumours would have taken more than a further week to have reached the 10 mm. diameter size.

### RESULTS

In this section, data on tumours of different types are presented separately except that the first section on skin tumours includes malignant skin tumours as well as non-malignant skin tumours. When interpreting the numbers of tumours found in the various groups one should bear in mind that during the period after the 75th week, when most of the tumours occurred, there were more group 3 (control) mice than group 2 (AC) mice alive and there were also more group 2 (AC) mice than group 1(FC) mice alive. This means that the occurrence of the same number of tumours of a particular type in the three groups does not imply the same incidence rate for that type of tumour in the three groups; it implies a higher incidence rate in group 1.

### *All skin tumours*

The cumulative numbers of deaths and skin tumours in the three groups are shown in Table III. There is a very clear difference between the skin tumour incidence in group 3 (control) mice (5/400 mice with tumours) and either the group 2 (AC) (207/400 mice with tumours) or the group 1 (FC) (208/400 mice with tumours), showing that treatment with either condensate predisposed to the development of skin tumours. Because of the poorer survival of group 1, the 208 tumours in group 1 corresponded to a significantly higher tumour incidence rate than the 207 tumours of group 2 ($P < 0{\cdot}05$ by the logrank test).

### *Malignant skin tumours*

As with " all skin tumours ", of which the malignant skin tumours form a part, the cumulative totals of lesions are displayed in Table III. The cumulative total in group 3 (control) (2 malignancies) differed markedly from those in either group 2 (AC) (93 malignancies) or group 1 (FC) (119 malignancies). In this case, however, there was already quite a large difference between group 1 and group 2 before allowance had been made for the worse survival of the mice in group 1, and this corresponded to an unequivocal difference between the two incidence rates. (Malignant tumours occur at a greater rate in group 1 (FC) than in group 2 (AC):

$P < 0{\cdot}01$ by the logrank test.) Fig. 1 illustrates an actuarial estimate of development of malignant skin tumours in mice of Groups 1 and 2 during the first 110 weeks of the experiment.

At post-mortem, 32 out of the 119 group 1 malignancies and 21 out of the 93 group 2 malignancies showed lymph node or distant metastases.

*Inflammatory ulceration*

During the later stages of the experiment 2 control mice, 38 group 2 (AC) mice and 52 group 1 (FC) mice developed inflammatory ulcers of the skin. These appeared most commonly near the centres of the treated areas of dorsal skin,

FIG. 1.—Actuarial estimate of percentages of mice without malignant skin tumours in groups 1 and 2 up to the 110th week of the experiment.

often in areas where all the hair follicles had already disappeared in response to treatment. Most such lesions were shallow ulcers that lacked the characteristic "roller" or "button" edge that suggests malignancy. Occasionally, their appearance or rate of growth gave rise to the suspicion of malignancy. All lesions which aroused such suspicion and a proportion of lesions which did not were examined histologically. A few [3 in group 1 (FC) and 2 in group 2 (AC)] of the former were found to be invasive carcinomas but none of the latter were. Histologically, the inflammatory ulcers showed atrophic changes with loss of pilosebaceous structures and excessive dorsal collagen in addition to infiltration by a wide variety of acute and chronic inflammatory cells.

*Lymphomas*

Of the 1200 mice in the experiment, 300 showed histologically confirmed generalized or localized lymphomas of various types at the time of death. Table IV summarizes the data on lymphoma incidence and shows that there were no marked or obvious differences between the three groups. Comparison of the incidence of individual types of lymphoma, *e.g.* generalized lymphoblastic, generalized lymphocytic, localized thymic, lymphomas localized to solitary lymph nodes to the spleen or to the genital tract (Thelma Dunn type A—see Dunn, 1954) also revealed no clear differences between the three groups. The comparison of incidence rates of lymphomas is difficult, because a lymphoma in a mouse that dies of some unrelated condition will be detected earlier than if the mouse lives until the lymphoma kills it. This means that the mice in group 1 (FC) would be expected, merely on the basis of their greater rate of death from other causes, to have a lymphoma detection rate greater than that of the mice of group 2 (AC), and group 2 (AC) greater than group 3 (control), and this was what we found, although the differences were so slight that they were not significant. There is, therefore, no indication that treatment with either type of condensate affects lymphoma incidence rates, in contrast to our findings in a parallel experiment

TABLE V.—*Lung Tumour Incidence*

| Group | Treatment | Weeks | Total deaths | Total with lung tumours | Grade* | | | | | Total with multiple tumours |
|---|---|---|---|---|---|---|---|---|---|---|
| | | | | | 1 | 2 | 3 | 4 | 5 | |
| 1 | FC ($T_2$ | 0–20 | 21 | 0 | 0 | 0 | 0 | 0 | 0 | 0 |
| | ×3 weekly | 21–40 | 40 | 1 | 1 | 0 | 0 | 0 | 0 | 0 |
| | in acetone | 41–60 | 39 | 1 | 0 | 1 | 0 | 0 | 0 | 1 |
| | | 61–80 | 77 | 7 | 3 | 1 | 3 | 0 | 0 | 2 |
| | | 81–100 | 131 | 22 | 10 | 9 | 3 | 0 | 0 | 8 |
| | | 101–120 | 92 | 26 | 9 | 13 | 3 | 1 | 0 | 9 |
| 2 | AC ($T_3$) | 0–20 | 8 | 0 | 0 | 0 | 0 | 0 | 0 | 0 |
| | ×3 weekly | 21–40 | 36 | 0 | 0 | 0 | 0 | 0 | 0 | 0 |
| | in acetone | 41–60 | 36 | 2 | 0 | 1 | 1 | 0 | 0 | 0 |
| | | 61–80 | 71 | 8 | 1 | 4 | 2 | 0 | 1 | 1 |
| | | 81–100 | 139 | 21 | 9 | 8 | 4 | 0 | 0 | 6 |
| | | 101–120 | 110 | 24 | 10 | 11 | 3 | 0 | 0 | 11 |
| 3 | Acetone | 0–20 | 8 | 0 | 0 | 0 | 0 | 0 | 0 | 0 |
| | ×3 weekly | 21–40 | 28 | 0 | 0 | 0 | 0 | 0 | 0 | 0 |
| | | 41–60 | 50 | 1 | 0 | 0 | 1 | 0 | 0 | 0 |
| | | 61–80 | 60 | 3 | 2 | 1 | 0 | 0 | 0 | 1 |
| | | 81–100 | 123 | 12 | 4 | 7 | 0 | 0 | 1 | 3 |
| | | 101–120 | 131 | 38 | 11 | 17 | 10 | 0 | 0 | 15 |
| Totals | | | | | | | | | | |
| 1 | F/CAcetone | Total | 400 | 57 | 23 | 24 | 9 | 1 | 0 | 20 |
| 2 | AC/Acetone | Total | 400 | 55 | 20 | 24 | 10 | 0 | 1 | 18 |
| 3 | Acetone | Total | 400 | 54 | 17 | 25 | 11 | 0 | 1 | 19 |

* *Grade 1* = benign non-invasive adenomas; *Grade 2* = tumours showing local invasion of surrounding lung and/or extension within airways; *Grade 3* = tumours that have replaced an entire lobe of the lung or have metastasized via the airways within the lobe in which they originated; *Grade 4* = tumours showing local extension beyond the lobe of origin (*e.g.* into chest wall, diaphragm or mediastinum); *Grade 5* = tumours that have given rise to distant metastases.

TABLE VI.—*Incidence of Neoplasms of Liver, Ovary, Mammary Gland and Subcutaneous*

| Group | No. in group | Average survival of animals | No. of animals with hepatomas | Time of death (weeks) of animals with hepatomas | No. of animals with ovarian neoplasms | Time of death (weeks) of animals with ovarian neoplasms | No. of animals with mammary carcinomas | Time of death (weeks) of animals with mammary carcinomas | No. of animals with s.c. sarcomas | Time of death (weeks) of animals with s.c. sarcomas |
|---|---|---|---|---|---|---|---|---|---|---|
| 1 | 400 | 77·6 | 8 | 84, 87, 88, 93, 98, 101, 103, 116 | 3 | 90, 95, 114 | 2 | 45, 88 | 5 | 73, 87, 95, 97, 112 |
| 2 | 400 | 81·2 | 6 | 93, 112, 112, 113, 114, 118 | 5 | 83, 84, 89 105, 111 | 4 | 79, 84, 97, 107 | 7 | 65, 80, 90, 100, 101, 101, 102 |
| 3 | 400 | 84·0 | 2 | 118, 119 | 6 | 90, 96, 103, 104, 110, 119 | 9 | 59, 70, 89, 93, 97, 101, 103, 108, 114 | 5 | 96, 98, 101, 107, 117 |

involving the application of the neutral fraction of tobacco smoke to the skin of mice (Roe, Kearns, Bishop and Peto, unpublished data).

*Pulmonary tumours*

The crude data on these tumours are presented in Table V. The observed numbers of pulmonary tumours in groups 1, 2 and 3 (57, 55 and 54 respectively) correspond to significant differences in detection rates (the expected numbers on the basis of equal incidence rates are 41, 51, and 74, respectively; groups 1 and 2 *versus* 3 $\chi^2 = 10{\cdot}6$: $P < 0{\cdot}01$). However, most pulmonary tumours in mice are relatively slow growing and may be present for a long time before they constitute a threat to life. Therefore, even more so than in the case of lymphomas, increased risk of early death from other causes is liable to favour the early detection of pulmonary tumours. Thus it is possible that the difference between the control group (group 3) and the two condensate-treated groups is a manifestation of earlier detection rather than of enhanced risk of pulmonary tumour development.

*Neoplasms of other sites*

The incidence of neoplasms of other sites in the three groups is shown in Tables VI and VII. Treatment with FC or AC had no obvious effect on the risk of development of parenchymal-cell hepatomas, ovarian or mammary tumours, subcutaneous sarcomas or neoplasms of a variety of other sites.

TABLE VII.—*Incidence of Neoplasms of Various Miscellaneous Types*

| Group | No. in group | Total miscellaneous tumours | No. | Details | Time of death (in weeks) |
|---|---|---|---|---|---|
| 1 | 400 | 10 | 1 | Thymic lympho-epithelioma | 15 |
| | | | 1 | Squamous carcinoma of forestomach | 107 |
| | | | 2 | Sarcoma of vaginal wall | 81, 81 |
| | | | 1 | Generalized spindle cell sarcoma | 86 |
| | | | 2 | Primary squamous carcinoma of lung | 80, 90 |
| | | | 1 | Exocrine carcinoma of pancreas | 61 |
| | | | 1 | Osteogenic sarcoma of lumbar spine | 77 |
| | | | 1 | Spindle cell sarcoma of the uterus | 107 |
| 2 | 400 | 6 | 1 | Primary squamous carcinoma of lung | 104 |
| | | | 1 | Sarcomatous polyp of uterus | 105 |
| | | | 2 | S.C. haemangioma | 74, 90 |
| | | | 1 | Malignant polygonal cell sarcoma of thymus with oesteoid changes | 108 |
| | | | 1 | Sarcoma in region of spine | 97 |
| 3 | 400 | 14 | 1 | Squamous carcinoma of forestomach | 119 |
| | | | 1 | Sarcoma of vaginal wall | 98 |
| | | | 4 | Sarcomatous polyp of uterus | 96, 104, 106, 117 |
| | | | 1 | Benign adenomatous polyp of large gut | 119 |
| | | | 1 | Haemangioma of spleen | 119 |
| | | | 2 | Adenocarcinoma of uterine cervix | 110, 114 |
| | | | 3 | Haemangioma of liver | 100, 101, 103 |
| | | | 1 | Intraperitoneal sarcoma | 118 |

### DISCUSSION

The results indicate that both types of condensate tested were actively carcinogenic for mouse skin. A consistent difference was detected ($P < 0{\cdot}01$ by the logrank test) between the rates of incidence of malignant tumours produced by the two condensates, and, mainly as a result of this, a difference between the overall rates of incidence of all types of skin tumours ($P < 0{\cdot}05$ by the logrank test), the group 1 (FC) mice suffering a greater tumour incidence rate than the group 2 (AC) mice.

Multiplicity of malignant skin tumours in individual mice, metastatic skin cancer and non-cancerous inflammatory ulceration also occurred more frequently in response to FC than AC.

The application of either condensate in acetone solution to the skin slightly but not significantly increased the rate of detection of malignant lymphoma, and significantly ($P < 0{\cdot}01$) increased the rate of detection of pulmonary tumours. The latter effect seemed more marked in FC-treated than AC-treated mice. However, especially in the case of pulmonary tumours, detection rates may not accurately reflect incidence rates, and all apparent differences may be artefactual. There was no indication that treatment increased the risk of parenchymal-cell tumours in the liver, ovarian or mammary tumours, subcutaneous sarcomas or other miscellaneous neoplasms as compared with mice given comparable treatment with acetone alone. The mechanism by which the application of the condensate to the skin could increase the risk of pulmonary tumours and lymphomas is uncertain. Absorption of carcinogens through the skin, or *via* the gastro-intestinal tract after licking, have to be considered as alternatives, but so has the possibility that the effect (if any) on lymphoma incidence reflects no more than a non-specific enhancement of the effects of a lymphoma virus present in the mice of the strain used. It is particularly noteworthy that no excess of neoplasms of the oral cavity, gastro-intestinal tract or urinary bladder was encountered.

To some extent the results are comparable with those reported by Day (1967). Mice of the same strain and sex were used and thrice-weekly treatments to the skin were continued throughout prolonged observation periods in both experiments. Furthermore, the condensates used were prepared in the same way. The incidence of mice with skin tumours (benign or malignant) and of malignant skin tumours seen in response to 60 mg. FC thrice weekly were seemingly considerably higher than that recorded by Day in response to 100 mg. or 50 mg. thrice weekly. However, mice in the present experiment survived better than those in Day's experiment, and calculations made after survival differences had been excluded indicated that risk of tumour development in response to similar doses was more or less the same (Lee, 1969, personal communication).

It is not possible to compare our data for internal neoplasms with those of Day, because he was concerned with such neoplasms only to the extent that they were a major or sole cause of death, whereas we recorded all neoplasms as revealed by a standard post-mortem procedure.

Unfortunately, as pointed out above (Materials and Methods section), the AC condensate studied was prepared from tobacco that did not resemble too closely air-cured leaf as normally incorporated into cigarettes. Nevertheless, the results indicate that, in case of a particular type of tobacco leaf, air-curing followed by bulk-fermentation did not markedly reduce the carcinogenicity of smoke condensate for mouse skin as compared with flue-curing followed by redrying. The reduction recorded, though statistically significant, was relatively small and difficult to equate with the sweeping claims made by Beffinger (1960).

As pointed out in the Materials and Methods section, the yield of condensate per cigarette was higher in the case of the air-cured, bulk fermented tobacco (AC) than for the flue-cured and redried tobacco (FC) [AC = 19·4 mg. per cigarette: FC = 16·1 mg.]. Treatment with 60 mg. of FC condensate therefore represented exposure to the smoke of more cigarettes than did treatment with 60 mg. of AC condensate. It seems likely that if the two condensates had been compared on the basis of exposure to particulate matter from the same numbers of cigarettes, no difference in carcinogenic effect would have been found.

An extensive quotation from Frankenburg's (1946) classical review on " Chemical change in the harvested tobacco leaf " deserves to be reproduced here lest anyone be tempted to regard the problems of producing a safer cigarette as simple or to regard results, such as those recorded in the present paper, as more than a possible pointer to what further research is desirable.

" An entire volume could be filled with a description of the various processes known as drying, curing sweating, redrying, ageing, resweating, and fermentation which have been developed for the manufacture of cigar, cigarette, and pipe tobaccos. Each of these operations has been adjusted to the special type and strain of tobacco used as the raw material, and is further modified according to the nature of the crop of a given " vintage ". In view of the divergence between the different, empirically developed methods of processing tobacco, it has been assumed, and also proved, that these different processes are not merely slight variations of one and the same basic schedule. On the contrary, in many cases the treatment applied to a given type of tobacco causes chemical changes which are opposite to those caused by the individual treatment of a second type of tobacco during its customary development to the finished product. It is, therefore, wrong and misleading to consider the chemical transformations which result from the processing of tobacco as identical or similar for the whole gamut of finished products. A study of the literature reveals that this misconception has often occurred, and that erroneous conclusions were reached as a result of it."

Many scientists, technicians, secretaries and others have assisted us in the work described in this paper and in the preparation of the manuscript itself. It is not possible to mention them all by name but we must express special thanks to Miss Janice Hotham and to members of the Histology Department of the Chester Beatty Research Institute.

We are grateful to the Tobacco Research Council, and particularly to members of the Planning Committee referred to in the introduction to the paper, for financing this project and advising at each stage of its prosecution.

The Chester Beatty Research Institute is supported by block grants from the British Empire Cancer Campaign for Research and the Medical Research Council.

### REFERENCES

BEFFINGER, I. J.—(1960) Memorandum entitled, ' Tobacco Smoking and Lung Cancer Research ' sent to Tobacco Manufacturers' Standing Committee in June, 1960, and circulated to a number of other Institutions and individuals at about the same time.

BENTLEY, H. R. AND BURGAN, J. G.—(1961) ' Cigarette Smoke Condensate Preparation and Routine Laboratory Estimation ', Research Paper No. 4. Tobacco Manufacturers' Standing Committee.

DAY, T.—(1967) *Br. J. Cancer*, **21**, 56.

DUNN, T. B.—(1954) *J. nat. Cancer Inst.*, **14**, 1281.
FRANKENBURG, W. G.—(1946) *Adv. Enzymol.*, **6**, 309.—(1950) *Adv. Enzymol.*, **10**, 325.
PENN, P. T. AND WEYBREW, J. A.—(1958) *Tobacco Science*, **2**, 68.